# Dialogic Feminist Gathering and the Prevention of Gender Violence in Girls With Intellectual Disabilities

**DOI:** 10.3389/fpsyg.2021.662241

**Published:** 2021-05-19

**Authors:** Roseli Rodrigues de Mello, Marta Soler-Gallart, Fabiana Marini Braga, Laura Natividad-Sancho

**Affiliations:** ^1^Nucleus for Research and Social and Educational Action, Department of Educational Theories and Practices (DTPP), Federal University of São Carlos, São Carlos, Brazil; ^2^Department of Sociology, University of Barcelona, Barcelona, Spain; ^3^Department of Pedagogy, Universitat Rovira i Virgili, Tarragona, Spain

**Keywords:** dialogic feminist gatherings, youth, adolescents, intellectual disability, gender-based violence, dialogic environment, prevention

## Abstract

Adolescent gender-based violence prevention and sexuality education is a topic of current concern given the increasing numbers of violence directed at girls. International organizations indicate that one in three girls aged 15 to 19 have experienced gender-based violence in their sexual relationships that this risk may be as much as 3–4 times higher for girls with disabilities. Following the good results obtained in the research project “Free_Teen_Desire” led by the University of Cambridge and funded by the Marie Curie Actions Program in the prevention of gender violence in adolescents through Dialogic Feminist Gatherings (DFG), the aim of study is to analyze Its transfer and impact on adolescent girls with intellectual disabilities. The DFGs are here understood as generators of a more dialogic environment for girls in general and we wonder if and how It is extended to the context of girls with disabilities. Thus, the research takes the form of a case study with a communicative approach on a DFGs. The intervention is carried out in a special school located in Valencia during the 2018–2019 and 2019–2020 academic years with a group of 19 non-mixed female students, female teachers, and the mother of one of the students. The study analyzes which are the transfer criteria to incorporate the DFGs in a special education context and what is their impact on the prevention of gender violence in girls with disabilities. The data collection techniques consist of two in-depth interviews, analysis of the field diary of 24 intervention sessions and a focus group with seven teachers. It is demonstrated that DFGs are successfully transferred to the special education context of the case study. The results show how contexts of safety, solidarity and friendship are generated which protect adolescent girls with disabilities from relationships with gender violence.

## Introduction

Gender-based violence is a growing problem in the 21st century, affecting young girls of all cultures and countries at increasingly younger ages. It is a problem that concerns even more women with disabilities who are more exposed to dependency, prejudice and marginalization and are at high risk of being abused by caregivers, family members, friends and others (Iudici et al., 2019). According to the latest World Bank (2019) report, “Violence against women and girls with disabilities,” it is estimated that globally, one in three women and girls with disabilities will experience gender-based violence in their lifetime. The results of a study funded by the WHO Department of Violence and Injury Prevention and Disability, considered children with disabilities as a high-risk group. Up to a quarter of children with disabilities (5% of children, about 93 million children) will experience violence in their lifetime, being three to four times more likely to be victims of violence than their non-disabled peers (Jones et al., 2012). It is known that almost one in three girls aged 15–19 years has experienced gender-based violence in their sexual relationships (World Health Organization, 2018). According to a World Bank (2019) report this risk could reach 3–4 times higher for girls with disabilities. A study of 2245 high school students in Sweden found that force at sexual debut (intercourse) is more common among adolescents with a disability (4.0%) than those not reporting any disability (1.6%), and is most common among those reporting multiple disabilities (10.4%) (Brunnberg et al., 2012). In the case of the World Bank (2019) report, the data show that the situation has not improved for girls and young women with disabilities, who today face 10 times more gender-based violence than women and girls without disabilities.

In this regard, evidence is growing on the importance of gender-based violence prevention education for people with developmental disabilities to increase opportunities for healthy sexual relationships and intimacy, promote positive sexual identities, and decrease the risk of sexual victimization (Murray, 2019). The school can be an ideal space to provide prevention and protection from gender-based violence and sexual abuse for children. To achieve this, there is consensus that interventions involving families, teachers, health agents, and community leaders are needed (Walsh et al., 2018; Elboj-Saso et al., 2020).

*Nothing About Us Without Us*, is the call of UN Women and the Committee on the Elimination of Discrimination against Women and the Committee on the Rights of Persons with Disabilities, which urges social, educational and health care entities to raise awareness that equality and sexual freedom for women and girls with disabilities depend on including their voices in feminist leadership to end the sexual violence they persistently experience. In this regard, studies identify the need to develop interventions to help women with disabilities recognize abuse and remove themselves from potentially abusive relationships and situations (Nosek et al., 2001; Skarbek et al., 2009). More recently, studies such as Iudici et al. (2019) highlight the importance of girls with disabilities having spaces for dialogue about these issues and the causes that provoke them. Thus, generating interactive spaces with a dialogic perspective is fundamental for the challenge of learning, in the topic we are considering here, of learning that allows them to identify, confront and prevent gender-based violence.

The consequences of experiencing gender-based violence in adolescent girls require further evidence. Research suggests that abuse and violence against women with developmental disabilities may exacerbate existing health problems or cause additional injuries. Psychological effects that have been identified include depression, anxiety, increased feelings of stress, and suicidal ideation. Negative physical effects of abuse also include physical harm and overall decreased physical functioning (intestinal, skin, and nutritional problems, as well as sexually transmitted diseases) (Hassouneh-Phillips, 2005; Plummer and Findley, 2012). According to international scientific evidence, one of the main barriers for people with disabilities is social exclusion and discrimination, which is an impediment to benefiting from the right to sexual education that prevents them from gender-based violence (Rohleder et al., 2019). This is a reality that is internalized as exclusionary in that socially it has been considered that people with disabilities do not decide for themselves on these issues.

Within the theoretical framework of preventive socialization of gender violence (Valls et al., 2008; Puigvert, 2014; Gómez, 2015; Puigvert, 2015/2016; Ruiz-Eugenio et al., 2020), dialogic feminism (Beck-Gernsheim et al., 2001; De Botton et al., 2005) and Dialogic Learning (Flecha, 2000) the Dialogic Feminist Gatherings (DFG) have been defined (Puigvert, 2016; Racionero-Plaza et al., 2018, 2020). The DFGs (Puigvert, 2016; Racionero-Plaza et al., 2018, 2020) are an educational action based on the preventive socialization of gender-based violence (Valls et al., 2008; Puigvert, 2014; Gómez, 2015; Ruiz-Eugenio et al., 2020). At its base is the understanding that one of the causes of the increase in gender violence at increasingly younger ages is a type of socialization that associates attraction to violence through a coercive discourse that is imposed among adolescents. One of the key aspects of the DFGs is the scientific content in violence prevention by giving the opportunity to contrast scientific evidence with the life experiences and reflections of the participants, allowing the participants, through dialogue, to build a collective knowledge that promotes relationships based on solidarity and favors the growth of healthy relationships that bring them well-being (Ruiz-Eugenio et al., 2020).

The DFG have demonstrated its effectiveness in the prevention of gender-based violence in adolescent girls without disabilities in diverse social contexts (Puigvert, 2016; Salceda et al., 2020). The DFGs create dialogic spaces for interaction where scientific evidence on the prevention of gender violence is analyzed, and where the voices of adolescent girls are empowered. The results of previous research show their incidence in establishing affective-sexual mental models in which attraction is linked to good treatment, friendship, equality and freedom. These spaces open opportunities to review these models and prevent from violent intimate affective-sexual relationships (Racionero-Plaza et al., 2018). In brief, DFGs generates interactive learning environments to foster learning, development, and relationships for adolescent girls with no special needs and that we analyze here whether it can be transferred to contexts of interaction between girls with special needs.

Thus, this research presents a case study developed in a Special School for the promotion of sexual health and egalitarian relationships in young girls with disabilities. The research analyzes whether interventions based on DFGs are effective to generate a dialogic interpersonal context with and for adolescent girls with intellectual disabilities and thus enhance learning to prevent gender violence, through a multidisciplinary professional intervention. To this end, the study focuses on two axes of analysis. First, to analyze whether the DFGs are transferable to special education contexts with adolescent girls with mild and moderate intellectual disabilities, as a context of dialogic interaction. Second, to know what successful results do teachers and educators perceive in the learning and relationships that make possible the prevention of gender violence in girls with intellectual disabilities.

## Materials and Methods

### Assessment Progress of the Case Study

The case we are analyzing is a unique case study, as it attempts to analyze in depth the improvements that DFG has carried out in a special school located in Valencia during the 2018–2019 and 2019–2020 academic years, with a group of 24 non-mixed female students with intellectual functional diversity between 15 and 24 years of age, students of a special school, seven female teachers and one mother. In the study, of the 24 girls, we had the informed consent of the guardians of 19 of them. All the teachers agreed to participate in the study, including the DFG coordinator; the school principal also agreed to participate in the study. The special school which is the context of study serves students from 3 to 24 years of age from the metropolitan area of Valencia. Its student body is diverse, including families of students belonging to low socioeconomic status and ethnic minorities. The school implements successful educational actions (SEAs), recognized by the international scientific community as interventions that ensure the best results despite the context in which they are implemented (Flecha, 2015).

Since the beginning of the implementation of SEAs in the special school, the teaching team participates in dialogic teacher training spaces that are contributing to the transfer of SEAs to the educational context (Roca et al., 2015). Participating in these evidence-based training spaces was the motivation to work in greater depth on violence prevention through the implementation of actions with impact. At the same time, the increased sensitivity to these issues promoted the educational team to detect more effectively the needs of their students, to face stories of abuse and to be aware of the need to bring scientific evidence closer to the students as their own prevention mechanism against violent affective-sexual relationships.

In the DFGs carried out in this special school, the participants are only girls, that is, they are not mixed, one of the options in which the DFGs are being carried out. This was chosen with the aim of favoring the creation of a context that could reinforce trust and support and, therefore, relations of solidarity among the participants. At the same time, the aim was to create a space that would include their voices, so that they could be heard on current feminist issues that affect their lives, following the dialogic feminist approach (De Botton et al., 2005) in which the DFGs are framed (Salceda et al., 2020).

In the case study, we have analyzed the DFGs carried out in the special school from February 2018 to June 2019. The periodicity with which they have been implemented has varied between weekly or biweekly. Due to the health pandemic crisis because of COVID-19 their operation has been adapted to the preventive measures dictated by official bodies, allowing them to continue to be performed to this day.

Dialogic feminist gatherings in this case are conducted outside of the regular class schedule, that is, during learning extension time (Flecha, 2015). The participants are of various ages, participate on a voluntary basis and are all women. The total number of participating girls with intellectual disabilities has been 19. Other educational agents, seven female teachers and one female family member of the center, have also participated.

The DFGs work like any dialogic gathering, considered as one of the successful educational actions identified by INCLUD-ED Consortium (2009). In the DFGs analyzed in the case study, each participant presents her interpretation of what is being worked on, motivating the collective creation of new knowledge from the interactions generated with the other participants. Its operation is based on the seven principles of dialogic learning (Flecha, 2000) in order to favor an egalitarian participation in which the maximum number of voices is heard. They are developed on the basis of scientific knowledge in the prevention of gender violence. Among the materials used we find several publications in Diario Feminista, a journal that disseminates a wide range of articles based on scientific evidence. Some examples we highlight are one that addresses female solidarity (Febre, 2018), another on new alternative masculinities (Uriarte, 2019) and on sexual education and full relationships (Garvín, 2019).

### Research Design and Data Collection Instruments

The case study has followed the principles of the communicative methodology that “emphasizes that egalitarian dialogues between researchers and the life world of the subjects under investigation are necessary to achieve higher levels of Social Justice” (Gómez-González et al., 2010). In the dialog with the research participants, the researchers present data and arguments about the issues based on the scientific production on the subject. In turn, the participants present their reflections and arguments based on their experiences in the life world. The interpretations of the reported situations and what the research indicates are being constituted and agreed upon by both parties (Gómez, 2019).

The communicative techniques used for the collection of empirical materials and the communicative analysis for this research were: two in-depth interviews, one with the director of the special school and another with the DFG coordinator; a focus group only with the female teachers participating in the DFG, and a field diary in which the coordinator of the DFGs recorded the interventions of the girls with intellectual disabilities participating in the meetings, the topics and the selected scientific content. The focus group in a communicative approach (Gómez-González et al., 2010) is organized from the natural group of people who already know each other; in the case studied, they were teachers who regularly participated in the DFGs and who freely agreed to participate in the research. As for the field diary notes, when necessary, the researchers asked the DFG coordinator for clarifications about the procedures, meetings, materials, and transcription of the speeches of the girls with special needs participating in the meetings.

Once the school agreed to participate in the research, all the adults who participated, voluntarily and freely, were informed of the research through an informed consent form. Anonymity and confidentiality were assured in the data collected and subsequently analyzed. The families of the girls with disabilities were informed through informed consent in order to agree to analyze their participation in the DFGs. The study was fully approved by the Ethics Board of the Community of Researchers on Excellence for All (CREA). Only the data referring to the participants who signed the informed consent form were used; the other data were discarded.

The results of the case study offer insights based on two central ideas: (1) the transferability of DFGs as a space for dialogic interactions to the context of special education and (2) evidence of the impact that DFGs are having on the lives of adolescent girls with intellectual disabilities, specifically, how they are promoting preventive interactions that can contribute to protecting these girls from gender-based violence relationships. Seeking to offer contact with the voices of the people involved in the DFG, in the results, literal statements made by them are brought in. It is important to do this both to triangulate the qualitative data collected, thus enhancing the validity of the qualitative study, and also with the need to make visible through the voice of the agents themselves the possibilities generated in the DFG.

### Data Analysis

The analysis of the information gathered from the transcripts of the two interviews, the focus group and the accounts recorded in the field diary, has been based on the two components of the communicative methodology: (1) the exclusionary dimension, which identifies the barriers that prevent transformation, since in the absence of these barriers, practices or social benefits would be available to excluded individuals or groups (Gómez-González et al., 2010) and (2) the transformative dimension, which includes the elements that overcome such barriers. Importantly, research on sexual violence prevention done with the communicative methodology shows that it is distinguished by its contributions to advancing the identification, prevention, and overcoming of gender-based violence (Puigvert, 2014), which can help health care providers and others caring for young women identify whether they have been victims of gender-based violence (Ruiz-Eugenio et al., 2020).

How to develop a dialogic space for girls with intellectual disabilities, in such a way that they feel supported and at ease to dialogue and to strengthen themselves regarding healthy affective-sexual and friendship relationships? In this question, about the transferability of the DFG to special education, the excluding dimension aggregates elements that hinder the participation and the establishment of trust in the group, and the transforming dimension aggregates the elements that had guaranteed the participation, trust, and learning of the participants in the dialogues.

What evidence of success demonstrates that girls with intellectual disabilities benefit from the interactive environment established in the DFG by learning to identify, protect themselves, and seek help when faced with abusive relationships? In this question, the exclusionary dimension aggregates elements that would prevent participants from appropriating tools to protect themselves and report sexual abuse, ensuring their freedom to be treated with respect, and the transformative dimension aggregates elements that ensure the appropriation of such tools, improving their own lives and the lives of others.

## Results

### Are Dialogic Feminist Gatherings, as Generators of Dialogic Context, Transferable to Special Education Contexts With Adolescent Girls?

In the case study, it can be verified that dialogical feminist gatherings are transferable to special education contexts. Director, DFG coordinator and female professors highlighted important elements for DFG to be successful in special education contexts with adolescent girls.

#### DFG Periodicity and Time

In the interview with the coordinator (INT1), she highlighted that the weekly meetings made it difficult for the girls to participate in DFG (the exclusionary dimension), since it is important to prepare oneself at home for the meeting by reading or watching the material combined for the dialogue. The change of the *meetings to the biweekly period* (transformative dimension) favored the preparation and, consequently, the participation of the students. In the same direction, the director (INT2) pointed out that she noticed the transference of the dialogue that is established in DFG to the family space, because the family is invited to help their girl to read – or to assist – the material that will be commented in the meeting (transformative dimension); according to her, such situation was more favored in the context of the confinement by the necessary isolation to face the COVID-19 pandemic, as it is observed in the passage below:

*“Yes, they take it home, the families have told us about it. In addition, during the confinement was a good moment to share DFG with families, mothers, grandmothers., they had the opportunity to participate, since at school this participation is more complicated.” (INT2).*

The positive effect of support and dialog with family members in preparation for participation in the meetings can be confirmed by comparing both attendance and participation in DFG in the 2018–2019 course and in 2019–2020, when the periodicity was changed to biweekly: The participation of girls in the dialogue has grown significantly (from 56 to 128 interventions). It can be interpreted that the change in the interval between meetings favored the time for the girls to receive support from someone in the house, to read the texts, highlight the excerpts they would like to comment on with the group, and dialogue with someone from outside the DFG. The interaction during the preparation, by guiding the attention (Rogoff, 1990) on the material and the subject and the dialogue generated coincide with what the theory on dialogic reading indicates: it is important for the increase of intersubjectivity, generating a better understanding of what is read and amplifying the reading of the world (Freire and Macedo, 1987). For girls with intellectual disabilities, this exercise becomes even more necessary.

About the time of the DFG, through the individual interviews, the principal and the DFG coordinator (INT2 and INT1) coincide in emphasizing that the DFG offers an extended study time, without mandatory participation as a factor favoring its transfer to special education (transformative dimension). The coordinator comments on how they reached this decision:

*“So, starting from this variety of groups to which they belong, we saw that it was the way in which more girls could participate and share this same space. We thought that outside school hours, so that they could participate freely in the school, and it was offered as a leisure and lunch time activity, and the truth is that we have had quite a good response.”(INT1).*

#### Group Composition

The director, during her interview (INT2), pointed out that the fact of being *only among girls* (transformative dimension) favored both the assistance and the participation in the DFG, because they felt comfortable to speak among women, being able to express their thoughts and expose intimate situations in the group. As for the teachers in the focus group (FG1) highlighted another aspect of the group composition that favored the transferability of the DFG to the group of adolescents with intellectual disabilities: the dialogue among diverse women (transformative dimension), that is, with different ages; students, professionals, and family members; women with and without disabilities.

*“And I think that it also changes the way they see us, because it was like, oh, and you also participate in the DFGs, well, that’s good, and they dared to tell you more things, because they think that we are people they trust to tell certain things, but after participating with them, yes” (FG1).*

As for the DFG as a space that contemplated the genericity of being a woman, but also the specificities of women with intellectual disability, the coordinator states:

*“I think it is important for them to talk about all their concerns, they are the same age but they also have interests, concerns, fears, but many times being young people with functional diversity they are not taken into account, they are infantilized, it is thought, society or the family, or even professionals, think that they do not think about these things, about the couple or motherhood and it is the other way around, when you start talking to them in an equal way you realize that they have the same concerns that you as a woman or that you also had at their age, or that you can continue to have. And I think it is important that they can also give their point of view and that we all know that they also have their thoughts, ideas and that they can contribute many things to society, if we give them a voice of course. If we don’t give them a voice, we can’t listen to their ideas, their points of view and how they see it” (INT1).*

The principle of equality of differences, of dialogical learning (Flecha, 2000), is clear here as a factor that acted in the transformative dimension of the transfer of the DFG to the context of special education with girls with intellectual disabilities. Being among different women constituted the dialogical environment where the specificities of each one (age, with and without disabilities, type of bond with the school) and their equality (being a woman) enriched the points of view and dialogues, making everyone learn from each other. The egalitarian dialogue (Flecha, 2000) between different and equal (Flecha, 2000) made it possible for a climate of trust to be established and for exclusionary factors such as prejudices and infantilization of girls with intellectual disabilities to be faced as barriers and overcome by all.

#### Climate of Trust and Female Solidarity

In the results, two elements stood out from the data as they coincided in the perception of the director (INT2), the coordinator (INT1), and the teachers (FG) (transformative dimension): (1) the establishment of a climate of trust and female solidarity as fundamental for the DFG; (2) the DFG as a space for the girls with disabilities to talk about their specificities, as girls with disabilities, and as a time for their genericity as women, being able to form and empower themselves for a more healthy and free sexual life of violence.

About the climate of trust among all in the activity, the coordinator explains how it was guaranteed by confidentiality and further strengthened the group (transformative dimension):

*“We have noticed in the second course that the group is more solid, in which we made it clear from the beginning that confidentiality was important, and in the first course there was a problem about talking outside the group and that was stopped, and this course we have noticed that it is more cohesive, even though the participants have varied.”(INT1).*

One of the teachers participating in the Focus Group details the climate of trust as a climate of female solidarity:

*“I think that creating this group of trust, of feminine solidarity, what we wanted was for them to know what feminine solidarity is and that we had to support each other, that’s what we are for, I think that having this safe space has been good for all of them.” (FG1).*

#### Type of Material to be Worked on in DFG

The scientific quality of the texts was also pointed out by the coordinator as transforming elements of the possibility of participation and support for girls. This element appears aggregated to the fact that the group received training on successful educational performance, gaining clarity on the fundamentals of giving access to students and families, including students with disabilities, to scientific knowledge (Rodriguez et al., 2020). This is also in preventing the quality of people’s health. In times of pandemic, when fake news and anti-scientific thinking demonstrate how they can threaten people’s health and lives (Buslón et al., 2020), DFG, which has as one of its characteristics basing dialogues on scientific knowledge (Puigvert, 2016), become even more necessary science-based professional training as well.

In the dialogic focus group with the teachers who participated in the DFG, they emphasized that the dialogue around the films and advertisements that the girls watch (transformative dimension) favored the participation and transformation of the girls’ perception about violent relationships and healthy relationships. One of the teachers said:

*“I think that precisely with this topic, with these students we have to put more emphasis because I think that they do not talk about what a relationship is, what you would like to have in a relationship, and then what they are left with is what they see in the movies, which may not be beneficial, for a relationship” (FG1).*

#### Issues in Dialogue

The topics chosen for the dialogue were drawn from those that are addressed in DFG with girls and women without disabilities, as they are issues that have affected all women around the world. Among the topics discussed were, for example, ‘friendships and romantic love as those who treat me well.’ These issues (transformative dimension) were pointed as fundamental to the transformation of the expectations and desires of women.

*“If, in relation to what you said, you arrived last year and saw how they reflected on it, that they don’t stay with what they see in the movies and so on. I think that there is a previous work, from the previous year when we started them, to see who you choose, how that person has to be, how that person has to treat you well, has to be your friend, there has to be communication, a dialogue between the two of you, to reach an agreement when you disagree, a person who shares your dreams. I believe that a lot of work was done in many discussions on the subject of choice, and on making attractive the one who treats you well, with a series of qualities, who listens to you, supports you in your decisions, and emptying of attractiveness what is sold to us in the movies, the typical chauvinist, sexist, violent, leader of the school. So we had to demystify this and see that it was really true” (FG1).*

#### Professional Team Training

The principal and the coordinator also indicated the professional team training (transformative dimension) as something that made possible the transfer of the DFG as an interactive and dialogic environment to the special school. About the previous training of the teacher in the subject, scientifically based, the director explains that she and other professionals of the school already had received it, because of their participation in the training in successful educational actions. The director affirms:

*“We started with successful educational actions, and when we saw that they were doing interactive groups, literary dialogic gatherings, we saw it as possible. Also thanks to participating in spaces such as Sherezade (women’s group that meets every month) or the seminar, a very important training space to think of ideas, projects or actions that can help students. So the DFGs came from my colleague who mentioned it at the school. We both participated in the seminar, and we thought it could be a good action to implement with the students at the school. And so, we thought of taking advantage of the time in the dining room to spend more time learning with the DFGs.” (INT2).*

Finally, both the coordinator (INT1) and the teachers recognize that the professionals learn from the DFGs and from the girls (transformative dimension). And that is an important factor for the success of the transferability of the DFGs to adolescent girls with intellectual disabilities.

### What Evidence of Success Do Teachers and Educators Perceive in the Prevention of Gender-Based Violence in Girls With Disabilities?

The interviews with the director and the coordinator, and the focus group with the teachers showed that the transformative dimension (36 mentions) of the DFG with girls with intellectual disability far exceeded the exclusionary dimension (7 mentions). But it was from the annotation in the field diary of the voices of the girls themselves that it was possible to clearly see the beneficial results of DFG for the prevention of gender violence by girls.

#### Dialogic Access to Information and/or Identification of Gender-Based Violence

According to the coordinator and teachers, the dialogic access to information allowed the girls participating in the DFG to identify gender violence in a general and specific way. The girls expanded their capacity to identify violence and prevent themselves against it, by viewing films and advertisements that naturalize violence and by dialoguing about it in DFG meetings. Thus, it is evident that the DFG constituted interactive learning environments, fostering learning, development, and relationships for girls with special needs.

The director explains the focus of the intended transformation with the offer of information and the realization of the dialogue around the themes:

*“What we pursue is that they have a voice, it is one of the issues we always talk about, that they have a voice within feminism, that women with disabilities have a voice, that they also transform themselves as women, that they are free women, that they have free relationships, that they know how to position themselves in the face of violence, that they know how to distinguish it when they suffer it, or that they position themselves when another partner may suffer it. We want all these aspects to be transformed in this direction. And, above all, their voice. This is an aspect that has always accompanied us in the transformation process. To listen to them, to know what disabled women have to say about all these problems.” (INT2).*

In the Focus Group, one of the teachers pointed out the change perceived in the participant girls from the DFG, based on dialogued information:

*“And then many dared to identify situations, because when we saw a TV ad and analyzed it, or texts that we have seen or real situations that have happened in current times, because they all watch TV they are on (Facebook and so on) and they know of cases that they know how to identify as cases of harassment or violence. So I think that to a certain extent, they have become aware of that situation, and they have dared to tell things. So I do think that this has been positive for them. And for me as a teacher, being part of a circle like this, and being able to share it with them. I think it has been very positive in general” (FG1).*

In the voice of the adolescents participating in the DFG, 19 mentions are found in the first school year that make the identification of gender violence and ways to prevent it a reality, and three times as many in the second school year. The following are some examples of the participants’ statements, which show their appropriation of the topic, concepts, and position taking.

– *“An unhealthy relationship can be a toxic relationship. A toxic relationship is arguing all the time, being bossed around.” (D1-P11)*
– *“I think it’s very good that silence did not happen to her to denounce, she is strong and brave.”(D1-P20)*
– *“Each one can choose her partner but carefully in case he hurts you.”(D2-P1).*
– *“To be brave means to step firmly with your feet on the ground and to put your mind at ease, to say: ’this is as far as I go, you are a bad guy.” (D2-P25).*
– *“Silence is not consent either: if it doesn’t tell me anything, it’s not yes either.” (D2-24)*
– *“I agree with girl 1 and girl 6, I find it disrespectful that they force a girl. That is not right.” (D2-18)*

#### Creation of a Dialogic Space Where Voice Is Given, and Silence Is Broken

The DFG has shown itself to be a space for dialogue, to give a voice to adolescents with intellectual disability, even breaking the silence about the violence suffered by them and by people they know. In this aspect, the director, coordinator, teachers, and family members recognize it, coinciding in their analyses. As for the participating girls, 19 manifestations in the first school year and 57 in the second school year reveal the power of DFG as a space for dialogue, to raise their voices, to denounce abuses.

In the teachers’ focus group, for example, they highlighted the DFG as a space to recognize and denaturalize violence, to dialogue, to listen to the voice of the girls, and to give them the opportunity to break the silence.

*“I would just like to emphasize the last thing that teacher A. said, I have seen that it has helped them not only as a space for trust and so on, but also to identify situations that they had somehow normalized in their daily life and took for granted, well it is not so much, and that it was really something serious and that they could suffer some danger because of what could happen to them, and they have identified it and have somehow taken it to their life, this is what I think is very important, because I have seen this. I think it is very important that they have a space of trust where they have been able to tell us about situations, but it has also helped them to identify situations that have occurred here. For example, the issue of advertisements. They have been able to identify “this should not be like this, this should not be allowed,” I think it is very important to emphasize it.” (FG1).*

As for the notes in the field diary, about creating a dialogic space, raising the voice and breaking the silence, several are the examples to be shown.

– *In the other school they didn’t treat me well. (D1-P15)*
– *To be safe is to be safe with whoever you are with: mother, father, partner, myself. They take care of me and hold me when I am afraid. I feel cared for at home and at school. (D1-P14)*
– *My E. used to touch me. (D1-P13)*
– *We learn in these gatherings and if we are doing well or badly. (D2-P2)*
– *Watching a TikTok program a boy picks up a girl to help her. (D2-P26)*
– *The woman appears lying down as if she were dead, as if she were a mannequin. (D2-P13)*
– *Women choose when they want to be touched. (D2-P6)*
– *If I come home and my boyfriend tells me to clean up I will tell him no. (D2-P24)*
– *It is disrespectful to force a woman. She has to be free. (D2-P18)*
– *I chose this phrase because of what it conveys, the girl has to give permission to do things if she wants to do them with the guy. (D2-P25)*

#### Trust, Solidarity, and Friendship

The establishment of a climate of trust, solidarity, and friendship at the DFG was not only a condition for the transfer of this action to a work context with adolescents with intellectual disabilities, but was also the result of the DFG, transforming relationships. In the date, we noted a mention made to this element in the exclusionary dimension, which refers to when there were, in the first school year, comments outside the DFG itself about what one of the girls said, generating distrust in the group. After agreeing on the need for confidentiality, the climate of trust was strengthened and also fostered solidarity and friendship among the girls.

The coordinator explains how the professionals of the school thought of the DFG as a space for dialogue, solidarity and development of friendship among the girls:

*“We think it can help them to have this climate of trust, a place where they can talk about issues that interest them. As well as we can discuss with them issues related to feminism and strategies that they may have. And then, we thought that they, for example, are in small groups during school hours, for example, they can be groups of 8–10 students. So we saw that this space could encourage them to get to know each other better and create this network of protection and friendship among them, since they don’t have groups as large as in high schools or universities. That is why we thought of a place where they could get to know each other and weave a friendship network that would help them to create new relationships among themselves and with the rest of their classmates” (INT1).*

The director exemplifies the result of trust as solidarity and friendship between girls and professionals as well:

*“For example, during the confinement, a student called me to share with me some messages she was receiving from a boy, a friend, who was telling her things she didn’t like on her cell phone. She had the confidence to tell me. I think that bonds of trust are created because we have discussed these issues. If this student had not talked about these issues, she would never have said to me, surely, “M., this is happening to me. This student is bothering me. On the other hand, if we discuss these issues with them and they know that they can talk about this with us. Then, if it happens to them they are able to share it.” (INT2).*

In the focus group, the teachers indicated the effective impact of the DFG as generators of solidarity and friendship networks among the girls, but also with the participating professionals, generating and consolidating an interactive and dialogic learning environment:

*“As she said, I have seen this network of female solidarity and friendship, even among them, supporting and advising each other, when someone told of a problem, how they offered to support her and to give her a solution. What we have all learned is a series of keys on how to act in case of possible harassment, if you are alone somewhere, what to do if you see something suspicious or who to ask for help. In other words, they have learned some tips and how to advise each other to support and protect them. So, even in the playgrounds, because they were from different classrooms, but then in the playgrounds they were also together and you could see this friendship. I didn’t even have any relationship with them because they are not my students, except for the delegates, who are not in charge of the subject and some of them are, but it is nice to see that they help each other, and if something happens in the playground, they come to me and tell me this has happened, that they have changed the way they look at us. We were part of this group because we were on an equal footing, and they knew it, on a level of trust, in which we can all contribute and help. And I remember that they came to me in the playground, and they didn’t go to look for another teacher, they came to me, and this is because it gives them security and confidence, because of the group that we have formed here, regardless of the position we have in the center” (FG1).*

And in the notes of the field diary, one can see how solidarity and friendship were being exposed by the girls:

– *The sisterhood (of women) reminded me of the club of the brave: they support each other, they defend each other, they stick together. (D1-P15)*
– *We talked about solidarity: helping women. (D1-P21)*
– *“Solidarity” as a key word in the discussion: that people help each other. (D1-P5)*
– *We have to go out as a group to feel protected. (D2-P25)*
– *I have had friends that I have given them advice: if he loves you he doesn’t have to hit you, he pampers you. (D2-P26)*
– *Solidarity, because at school we have learned to be supportive. (D2-P12)*
– *I have chosen the equality of all people. We are all equal in rights, but we are also different in character, attitude,. (D2-P18)*
– *Network of women united for education: I think it is good that women have opportunities to work and study because women are capable. (D2-P5).*

#### Coercive Discourse

The recognition of the existence of coercive discourse about girls, and on them, was another aspect that emerged as evidence of transformation in the coordinator’s interview and in the notes of the field diary, coinciding with previous research on DFG with girls without disabilities (Racionero-Plaza et al., 2018, 2020). As an excluding dimension, the coordinator pointed out how girls sometimes give in to the coercive discourse that naturalizes gender or sexual violence as normal in relationships and, as a transforming dimension, she highlights when girls identify that they are pressured to naturalize such violence:

*“Yes, I think that the DFGs do help, with the dialogues that take place, to identify it. It is true that they are able to say it, but it is also true that although it is noticeable, at a certain moment the coercive discourse weighs a lot, that is why I think we have to continue working more. I don’t know, it is like during the 2 years we have been talking, yes, we are talking, but I think that I hope it will be with them for a long time, because I am sure they will have experiences in which they will have to make decisions. This happens to us, for example, with the attraction to violence, sometimes you see in yourself that you can have it clear, but then it happens to you and you say to yourself “it has already escaped me.” Well, I see the same thing happening to them. In affective-sexual relationships, even in friendships, they are very much into being a pimp, they are always waiting to see what they are told., it’s like they are very clear about the theory but then it’s hard for them on a day-to-day basis.” (INT2)*

In the field diary, notes refer to the girls’ recognition of situations in which the discourse is coercive and they are opposed to it, showing the impact of the DFG. It is worth mentioning that all the manifestations appear in the second school year, that is, after the practice of at least one school year. One of those is highlight here:

– *They can’t force you to get married either, what do you think? (D2-P3)*

#### Transference to Everyday Life

The transference of the learning done in the DFG to daily life was another aspect that was revealed in the analysis of the data. In the focus group of teachers and family members, the lack of support networks among friends and with the family as an obstacle to the transference of learning emerged as an exclusionary dimension.

*“As for the link to extrapolate what they learn here, I think it depends a lot on the context they have outside. If they have a secure network of friends, of true friendships, they dare to do these things, to continue with what we do here. Then there are cases in which, if they don’t have that safety net, both family and friendships in their closest environment, they recognize it themselves” (FG1).*

In the notes of the field diary, 58 participations were identified that demonstrate that the girls transfer what they learn in the DFG to other spaces of their daily life. Some examples are:

– *“We have to be careful about what we upload to Facebook: like videos of violence. (D1-P3)*
– *We don’t post anything on Facebook about our intimacies. (D1-P14)*
– *I went to a talk about gender violence. It is not only physical but also psychological. (D2-P1)*
– *When we go shopping alone, we keep an eye out for any guy. The four of us together can make a shield, but we go with fear. (D2-P25)*
– *I wish there were more resources in my neighborhood community to prevent sexual harassment. (D2-P13)*

#### Increased Participation in Other School Spaces

It was an aspect that emerged in the data, but little was mentioned. In the focus group and in the field diary, they coincide in recognizing how some girls have shown a change after participating in the DFGs with respect to an improvement in their safety, which in turn has been seen in an increase in their participation in other school spaces, in some of the girls, although this aspect is one of the least mentioned. For example, in the focus group the teachers explain how they observed this with two students:

*- “P7 now participates more in the rest of the gatherings, perhaps the DFGs have given her the support and security she needed to intervene in other educational actions.” (FG1)*

*- “as a result of participating in this I saw that she proposed more and it caught my attention, because I thought look at this little girl, she does reason, and maybe this has helped her to find her space.” (FG1)*

In the field diary, regarding this category, two of the girls coincide in pointing out that by strengthening the network of support and solidarity, DFGs make them more involved in other school settings:

*- “About freedom-defend: In the playground we can help people who are crying, solve the problem and talk to them.” D2-P2*

*- “There is a brave club, I am brave because I help my classmates.” D2-P14*

#### Romantic Love and Friendship Related to Those Who Treat Me Well

It is another of the most recorded themes in which almost all are annotations analyzed as transforming. This topic also comes up in the focus group with the teachers who participate in the DFG, highlighting how this space provides girls with the opportunity for transformation:

*“I said, what relationship I want in my life, if it brings something to me, if it doesn’t bring anything to me, and so I chose to distance myself from certain people. And the truth is, it was the first one and I said to myself, there are still people older than me, who do not have that clear in life, and here they said it with such simplicity, with such naturalness, that I was overwhelmed. And that’s why I said, we have to keep doing it, and in fact I started to take some high school students, in fact later they started to participate, and jeez it’s very nice, to see how they start this path and then continue working, because then they analyzed ads, and saw the non-egalitarian attitudes. and how they perceived it, and how they denounced it, which is true as S. says, in a safe environment, but jeez, it’s very hopeful.” (FG1)*

And another teacher adds:

*“no, that romantic love from the movies that still have it. So I really enjoyed taking the leap into DFGs to see that change, no. But I can see that those reflections, at least, are on the way, toward changing that, and toward having much healthier and more affectively satisfying relationships. Because they always focus more on protecting them, on instructing them, and so on accompanying them, on creating those affective bonds. And I see it from the outside, not knowing them, and suddenly seeing such authentic approaches, saying, how nice that they are getting to think like that and transforming them as well.” (FG1)*

This is highlighted mainly in the field diary, where there are records from the girls that reflect transforming messages that are part of the dialogues on ideal love and well-mannered relationships, which are internalized by the girls and become a guide for the relationships they are building and long for in their lives. In the diary there are 43 transforming entries, compared to three excluding ones.

In the girls’ interventions, love is identified as a feeling that is awakened only with good treatment and respect, separating it from deceit or mistreatment. In addition, it is not only identified in intimate relationships, but also awakens in those close to them, such as family or friends:

*- Love is to love: family, friends, and partner. (D1-P13)*

*- To love a person is that he/she is not just for you that person. (D1-13)*

*- Love is friendship, generosity, sharing with those who love you,. (D2-P18)*

*- It’s not a true friendship if he/she cheats on you. (D2-25)*

*- It is also the day of friendship, freedom and love (St. Valentine’s Day)/D2-P21)*

*- Men and women have to agree to treat each other well. (D2-P25)*

*- If you cheat on me or insult me it is not love. (D2-P14)*

*- It reminds me of what happened to me on a field trip, a classmate who said that if I slept with him, I said no because I didn’t trust him, that I wouldn’t allow him to do that. I left crying and went with a friend. (D2-P2)*

#### Change in the Professionals’ Recognition That Girls Can Do More

This is another aspect of the case study analysis that shows how the DFGs have allowed, by being set up as an interactive learning context, a change in the way girls are viewed, as they have seen that girls are more capable than they expected.

*“And then I told my boyfriend about it, oops a super cool thing happened to me, because sometimes it seems like we don’t know to what extent they understand us, right? But they do understand us because then they are able to act in the right way, and to identify it, as A. and A. were saying. For that part, they are useful to them.” (FG1)*

It is also seen that participating in the DFGs has led to a change in the way teachers look at girls, who see them in a different light.

*- “And I was very surprised because I remember that I, especially, in a gathering that we had on Valentine’s Day, I was very surprised because I realized that, in fact when I left here I talked about it with a friend of mine, I told her, I wish you could have come and witnessed what I experienced, because I say it and I get excited, because for me it was very emotional and very hopeful.” (FG1)*

*- “It happens to me like A, being from Physical Education, to me it has changed the way I look at them, because without wanting to, in Physical Education you do not have time to deal with certain issues, not to talk with them about certain things, and when participating in the DFG they left me hallucinated in terms of the reflections they made.” (FG1).*

This change of outlook has involved the teachers learning from the girls.

*- “And I said: if we teachers are supposed to guide a little bit, and I am almost learning more from them than they are learning from me. I also liked this aspect, because in the end the DFGs are productive for everyone, not only for the students, but also for the teachers, because we also understand how to see the reality of the adolescents.”(FG1).*

One teacher explains that the DFGs have allowed them to learn together, to learn from each other.

*- “I was going to say along the same lines, and that really I, for example, the students who are in the transition to adult life course, I had them in high school, and the SEAs started in their group, and there we began to change the world, we also learned together, to see other visions, other cultures, and I learned a lot from the Roma ethnic group, and the step, it depends on the culture in which you are, because I sincerely believe that I can advise from the culture in which I have been raised.” (FG1).*

Finally, we highlight the remarkable growth of participation of girls, exposing their ideas, from one period to the next in the DFG. This demonstrates how the DFG has effectively constituted a context of dialogical learning for them. Analyzing the frequency of participation with speech in the two school years, it was possible to verify that of the 19 participants who attended the DFG in the two school years, 12 showed increased interventions in the group, making their voices heard. This means that 63% of the participants increased their participation in this space of dialogue among women.

## Discussion

According to international scientific evidence, one of the main barriers for people with disabilities is social exclusion and discrimination, which is an impediment to benefiting from the right to sexual education that prevents them from gender-based violence (Rohleder et al., 2019). This is a reality that is internalized as exclusionary in that socially it has been considered that people with disabilities do not decide for themselves on these issues. However, the case study shows that when this dimension is transformed, creating a space for context of dialogic relationships (Flecha, 2000), these adolescent girls with intellectual disabilities benefit from mechanisms that are effective in preventing violence.

Therefore, our data invite further research on how DFGs can be an educational intervention aimed at responding to the existing gap in this field, given the lack of programs in sex education and violence prevention with adolescents and young people with disabilities (Murray, 2019). These are encouraging data since evidence shows that adolescents and young people with disabilities are exposed to a high risk of being victims of sexual violence in their different developmental environments (Jones et al., 2012; Iudici et al., 2019), especially young women with intellectual disabilities. The results we present aim to contribute to reduce the risk to which adolescents and young women with intellectual disabilities are exposed to suffer violence in their affective-sexual relationships, while at the same time helping them to build healthy relationships that bring them well-being.

In addition, previous studies show that, despite the high risk of suffering violence, there are a low percentage of complaints due to the fact that victims with disabilities encounter numerous barriers in their environments to report (Iudici et al., 2019). UN Women, the Committee on the Elimination of Discrimination against Women and the Committee on the Rights of Persons with Disabilities point out, from international recommendations, the importance of including the voices of adolescent girls and young women with disabilities in order to seek their involvement in all parts of the process of ending the sexual violence they suffer. In this way, the empowerment of those who suffer violence is pursued with the training of skills that prevent them from violent relationships. Creating learning environments and interactions for girls in special education is one way and the DFGs are a space for affective-sexual education.

The data obtained in the case study shed light on two aspects. On the one hand, it identifies which elements have been favorable in the transferability of the DFGs to a special education context with adolescent girls and young women, as well as the conditions that have provided the successful results that have been obtained. And on the other hand, the improvement perceived by teachers and educators in the prevention of gender violence in girls with disabilities through the implementation of the DFGs.

We start from previous studies that have already shown how interactive environments based on dialogic learning create a framework that enhances learning by improving the results, while protecting them from any kind of violence (Flecha, 2000, 2015). In special education settings the results have been also corroborated, showing that creating interactive learning environments in special education is beneficial, not only in students with disabilities, by improving the quality of learning received, but also in the professional progress of teachers (García-Carrión et al., 2018).

The benefits achieved are obtained by transforming the interaction patterns (Aguilera-Jiménez and Prados Gallardo, 2020) of a traditional classroom into ones that create conditions that favor the creation of learning environments based on solidarity and mutual support. These benefits can be reflected both in the increase of supportive interactions either in the group itself or beyond the school, in the improvement of communication skills and instrumental learning, the improvement of relationships among students (García-Carrión et al., 2018). The data from our research show that DFG generates a dialogic environment in the school and manages to connect girls with intellectual disabilities with their environments, whether immediate, such as the family or the school, or other non-immediate environments in which they develop (social networks, their community of neighbors, their neighborhood or city where they live).

As already mentioned, the successful program of the DFG (Puigvert, 2016; Racionero-Plaza et al., 2018, 2020) is built on the theoretical framework of preventive socialization of gender violence (Valls et al., 2008; Puigvert, 2014; Gómez, 2015; Puigvert, 2015/2016; Ruiz-Eugenio et al., 2020), dialogic feminism (Beck-Gernsheim et al., 2001; De Botton et al., 2005) and Dialogic Learning (Flecha, 2000). This is an educational action that is giving positive results with adolescents without disabilities in the prevention of gender violence (Salceda et al., 2020). This is a priority objective given the evidence showing the impact of the first love learning on the rest of future relationships (James et al., 2000).

Something else that is apparent in the data we have analyzed in the case study is with respect to coercive discourse. Recent analyses show that one of the key components of gender violence victimization among adolescents is the influence on the preferences of adolescent girls to start having an affective-sexual relationship. These preferences are strongly influenced by a coercive discourse that reproduces in the peer group interactions that pressure and push them to have relationships with violent boys (Racionero-Plaza et al., 2021). In this regard, the way of talking to friends and peers can influence preferences, including the different models of masculinity, which is important since each model of masculinity plays a different role in perpetuating or eliminating gender violence (Flecha et al., 2013). Therefore, to prevent gender violence among adolescents, it is crucial to promote communicative interactions that contribute to changing the preferences of girls toward a model of masculinity away from gender violence, and that eliminating the pressure exerted by the coercive discourse in the peer group. In addition to generating interactive learning environments, it is necessary to ensure that these environments are concerned with promoting the identification of coercive discourses and decision making for sexual relationships free of violence. In our analyses, we have observed that thanks to the participation of girls in the DFGs, it has been possible to promote communicative interactions that promote a discourse in the peer group that not only enables them to recognize coercive discourse when it appears, but also to express their opposition to it.

## Some Considerations to Continue

From our results we can conclude that, in the experience developed, described, analyzed and discussed, the DFGs proved to be transferable to the context of special education, generating dialogic interactions that improve the learning and relationships of girls with intellectual disabilities in terms of sexual education. The overall form of the DFGs was maintained; although there were changes resulting from the dialogue with the girls themselves (use-enders) regarding the frequency of the meetings and their duration, the content discussed was the same as that developed in DFG with girls without disabilities, i.e., central themes for affective-sexual choices, based on scientific texts and sources.

Finally, due to being a single case study, we consider important to replicate it in other contexts in order to gather further evidence on the effective transferability of the DFGs to promote a dialogic learning environment in diverse types of educational settings and as a promotion of a dialogic learning environment.

## Data Availability Statement

The raw data supporting the conclusions of this article will be made available by the authors, without undue reservation.

## Ethics Statement

The studies involving human participants were reviewed and approved by Ethics Board of the Community of Researchers on Excellence for All (CREA). Written informed consent to participate in this study was provided by the participants’ legal guardian/next of kin.

## Author Contributions

RM, MS-G, FB, and LN-S conceived and designed the study. LN-S conducted a preliminary analysis of the data. RM and LN-S completed the data analysis, checked, cleaned, and tabulated the results. RM and LN-S drafted the first version of the manuscript. RM, MS-G, FB, and LN-S have contributed to revising the manuscript and have approved the final version of the manuscript. All authors contributed to the article and approved the submitted version.

## Conflict of Interest

The authors declare that the research was conducted in the absence of any commercial or financial relationships that could be construed as a potential conflict of interest.
